# Mint3 as a Molecular Target Activated in the Early Stage of Hepatocarcinogenesis

**DOI:** 10.3390/ijms26041430

**Published:** 2025-02-08

**Authors:** Masaki Nishitani, Hikari Okada, Kouki Nio, Tomoyuki Hayashi, Takeshi Terashima, Noriho Iida, Tetsuro Shimakami, Hajime Takatori, Masao Honda, Shuichi Kaneko, Takeharu Sakamoto, Taro Yamashita

**Affiliations:** 1 Department of Gastroenterology, Kanazawa University Graduate School of Medical Sciences, Kanazawa 920-8641, Ishikawa, Japan; nishi86masa68@gmail.com (M.N.); okada0922@gmail.com (H.O.); nio@m-kanazawa.jp (K.N.); tohayashi@m-kanazawa.jp (T.H.); tera20jp@gmail.com (T.T.); niida@m-kanazawa.jp (N.I.); shimakami@m-kanazawa.jp (T.S.); takatori@m-kanazawa.jp (H.T.); mhonda@m-kanazawa.jp (M.H.); shuichikaneko@gmail.com (S.K.); 2 Department of Cancer Biology, Institute of Biomedical Science, Kansai Medical University, Hirakata 573-1010, Osaka, Japan

**Keywords:** hepatocellular carcinoma, munc18-1-interacting protein 3, hypoxia-inducible factor-1

## Abstract

Mint3 enhances aerobic ATP production with subsequent nuclear translocation of hypoxia-inducible factor-1 (HIF-1) and activation of angiogenesis-related genes. It remains unclear if and when Mint3 is activated and whether it is involved in hepatocarcinogenesis. We explored the expression of Mint3 in surgically resected hepatocellular carcinoma (HCC) tissues. We evaluated the effects of Mint3 knockdown on spheroid formation capacity and subcutaneous tumor growth in immune-deficient mice. We used Mint3 knockout mice to evaluate the effects of chemically induced HCC development. Mint3 was overexpressed in well-differentiated HCC with the activation of HIF-1 target genes irrespective of the absence of hypervascularization. Mint3 knockdown ameliorated the expression of HIF-1 target genes in patient-derived HCC cell lines and suppressed spheroid formation. Mint3 knockdown further inhibited subcutaneous tumor formation in vivo in immune-deficient mice. Chemical HCC development induced by N-nitrosodiethylamine (DEN) or DEN/CCl4 was dramatically suppressed in Mint3 knockout mice compared to control mice. Mint3 plays a crucial role in early-stage HCC development before hypervascularization by activating HIF-1 target genes before the tumor becomes hypoxic. Mint3 is a molecular target that prevents HCC development in the early stages.

## 1. Introduction

Hepatocellular carcinoma (HCC) is the second most lethal cancer after pancreatic cancer, with a 5-year survival rate of 18% in the world [1,2]. HCC generally develops in the background of liver fibrosis and cirrhosis caused by viral hepatitis, alcohol consumption, and fatty liver diseases caused by lifestyle [3,4]. The incidence of HCC continues to increase, especially in Western countries [5]. HCC is considered to develop through a multistep process that progresses from regenerative to dysplastic nodules and well-differentiated HCC without hypervascularization, and then to HCC with a hypervascular nature [6,7]. Hypervascularization of tumors is generally caused by hypoxic conditions and subsequent activation of a transcription factor called hypoxia-inducible factor-1 (HIF-1). HIF-1 transcriptionally activates various target genes, including vascular endothelial growth factors (VEGFs) [8,9]. Indeed, VEGFs and their receptor VEGFR2 are activated in HCC; therefore, the major molecular targets are inhibited by various receptor tyrosine kinase inhibitors, such as sorafenib [10], regorafenib, lenvatinib [11], and cabozantinib [12], and monoclonal antibodies, such as bevacizumab [13] and ramucirumab [14], for the treatment of patients with advanced-stage HCC [15]. However, the molecular targets activated in the early stages of hepatocarcinogenesis before the activation of angiogenic signaling remain elusive.

The Warburg effect is a phenomenon in which cancer cells perform aerobic glycolysis rather than oxidative phosphorylation even in the presence of sufficient oxygen [16]. One potential benefit of the Warburg effect in cancer cells is the high adaptability of cancer cells to hypoxic conditions, which may be closely associated with the invasion and metastasis of cancer cells. HIF-1 plays a fundamental role in the adaptation of cancer cells to hypoxia by activating genes involved in aerobic glycolysis, such as glucose transporter (GLUT), hexokinase 2 (HK2), and VEGFA [17]. However, the Warburg effect was observed in cancer cells without hypoxic conditions, suggesting that cancer cells perform aerobic glycolysis before exposure to hypoxia and subsequent activation of HIF-1.

HIF-1 is composed of α and β subunits, and its transcriptional activity is negatively regulated by factor inhibiting HIF-1 (FIH-1) via hydroxylation of HIF-1α [18,19].

Munc18-1-interacting protein 3 (Mint3, also known as amyloid beta precursor protein binding family A member 3) is a member of the X11 protein family [20]. We previously demonstrated that Mint3 inhibits the suppressive activity of HIF-1 by FIH-1 [21]. Therefore, Mint3 overexpression results in excess binding to FIH-1, releasing HIF-1, and activating the HIF-1 transcriptional program without hypoxia [22,23]. Mint3 promotes tumor progression in several cancers, including breast, pancreatic, and bladder cancers [24,25,26,27,28,29]. In breast cancer, Mint3 enhances the Warburg effect by activating HIF-1 during normoxia [27,29]. However, it has not yet been clarified whether Mint3 is activated in HCC or its relationship with the hypervascular nature of the tumor, which is a hallmark of classical HCC diagnosis. In this study, we evaluated the expression and role of Mint3 in hepatocarcinogenesis in mice and humans. We further evaluated the potential of Mint3 as a molecular target for preventing HCC development.

## 2. Results

### 2.1. Mint3 Is Overexpressed in Well-Differentiated HCC

**Figure 1:**
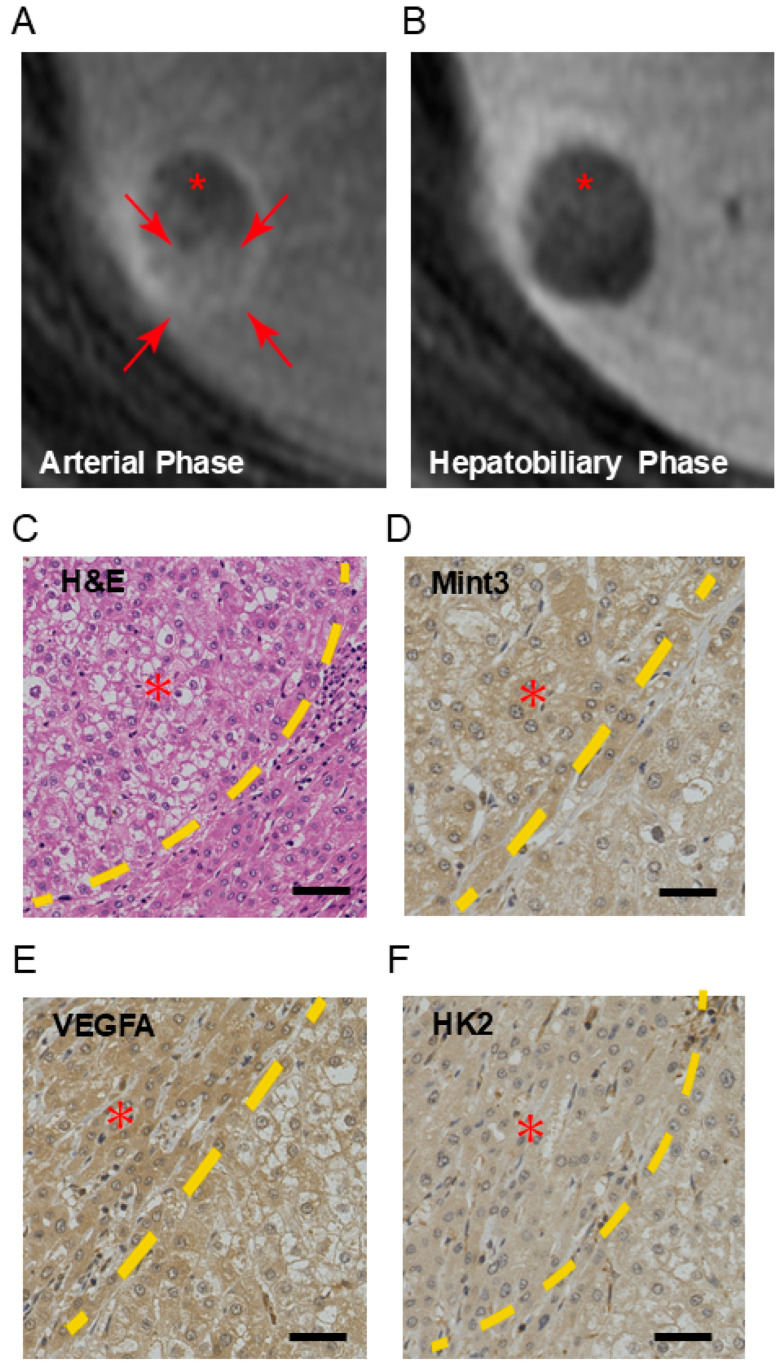
Expression of Mint3 and HIF-1 target proteins in hepatocellular carcinoma presenting a nodular appearance. (**A**,**B**) Gd-EOB-DTPA-enhanced magnetic resonance images: (**A**) arterial phase; (**B**) hepatobiliary phase. Red arrows (**A**) indicate hypervascular region in the nodule. Red asterisk (**A**,**B**) indicates hypovascular region with mild uptake of Gd-EOB-DTPA detected at hepatobiliary phase. (**C**) Microscopic image stained with hematoxylin and eosin. (**D**–**F**) Immunohistochemistry analysis of Mint3 (**D**), VEGFA (**E**), and HK2 (**F**) expression in HCC. Yellow dotted line indicates the boundary line between well-differentiated HCC and moderately differentiated HCC. Red asterisk (**C**–**F)** indicates well-differentiated HCC region. The ratio of staining intensity of well to that of moderately was slightly higher in well-differentiated HCC. Black scale bars in microscopic images indicate 100 μm.

We evaluated the expression of Mint3 in nodule-in-nodule HCC, where part of the HCC showed hypervascularity in response to cell proliferation and subsequent hypoxia. MRI findings clearly showed a hypervascular region (* indicated in Figure 1A,B) in the periphery of the hypovascular nodule. Histologically, the hypervascular region showed moderately differentiated HCC, whereas the hypovascular nodule showed well-differentiated HCC with clear cell and fatty changes (Figure 1C). The ratio of staining intensity of well-differentiated HCC to that of moderately differentiated HCC in Mint3 was 1:1.14.

We evaluated the expression of HIF-1 target proteins such as VEGFA and HK2 by IHC in both the hypovascular and hypervascular regions (Figure 1D–F). The ratio of staining intensity of well-differentiated HCC to that of moderately differentiated HCC in VEGFA was 1:1.25, and in HK2 was 1:1.11. Interestingly, Mint3 expression was slightly higher in the hypovascular well-differentiated HCC region compared with the hypervascular moderately differentiated HCC region, suggesting its role in inducing HIF-1 targets.

We further evaluated the expression of Mint3 and downstream targets in two additional nodule-in-nodule HCCs and an early well-differentiated HCC in the surgical specimens, all of which showed similar staining patterns (Supplementary Figures S1–S3).

We investigated the expression of Mint3 in surgically resected 100 HCC tissues and evaluated the expression patterns of Mint3 in terms of clinicopathological findings (Table 1). Mint3 expression was not associated with age, sex, etiology, liver cirrhosis status, or tumor size, but was associated with low serum alpha-fetoprotein (AFP) levels (*p* = 0.029) and well-to-moderate histological grade (*p* = 0.013) with statistical significance. AFP levels and histological grade were the only parameters associated with Mint3 expression. These data suggest that Mint3 plays a role in early-stage HCC development, with histological features of well-differentiated morphology without hypervascularization.

### 2.2. Downregulation of Mint3 Inhibits HCC Growth In Vitro and In Vivo

**Figure 2:**
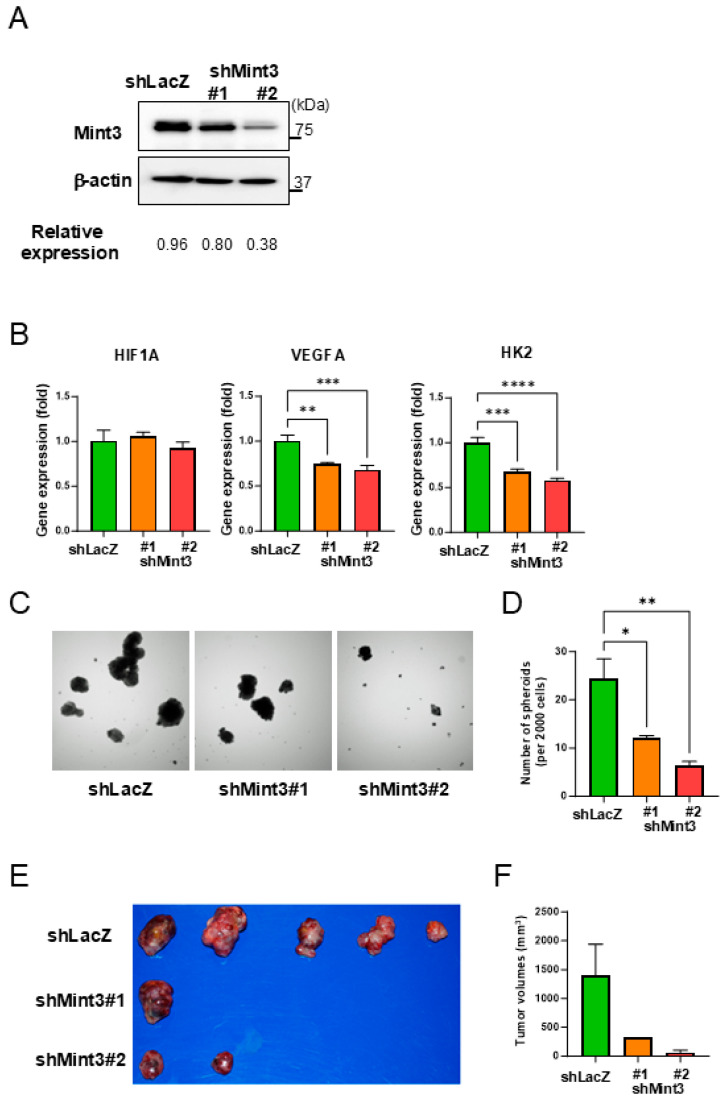
Suppression of Mint3 expression attenuates expression of HIF-1 target genes and tumorigenicity in well-differentiated HCC patient-derived KH cells. (**A**) Western blot analysis of Mint3 expression in control (shLacZ) and Mint3-suppressed (shMint3#1 and #2) KH cells. The results of quantification of blots were defined as the relative expression. (**B**) qRT-PCR analysis of HIF1A, VEGFA, and HK2 in control and Mint3-suppressed KH cells. ** *p* < 0.01, *** *p* < 0.001, **** *p* < 0.0001. (**C**) Representative photographs of spheroids in control and Mint3-suppressed KH cells. (**D**) Number of spheroids per 2000 control and Mint3-suppressed KH cells. * *p* < 0.05, ** *p* < 0.01. (**E**,**F**) The tumorigenic capacity of control and Mint3-suppressed KH cells in subcutaneous xenograft models using NOD/SCID mice. (**E**) Photos of tumors at the endpoint. (**F**) Median tumor volumes of control (n = 5) and Mint3-suppressed KH cells (shMint3#1; n = 5, shMint3#2; n = 5). Statistical analysis could not be performed because only one (shMint3#1) and two (shMint3#2) subcutaneous tumors developed in NOD/SCID mice.

To evaluate the role of Mint3 in HCC, we used patient-derived HCC cells (KH) previously established from well-differentiated HCC tissues with preserved hepatic gene expression patterns. We established three KH cell lines (control shLacZ, shMint3#1, and shMint3#2) using a lentiviral system and performed quantification as the relative expression. Relative expression of control was 0.96, shMint3#1 was 0.80, shMint3#2 was 0.38, and shMint3#2 was most strongly suppressed. Accordingly, we confirmed weak (shMint3#1) and strong (shMint3#2) Mint3 knockdown compared to control cells (Figure 2A). The expression of HIF-1 target genes VEGFA and HK2 was suppressed by Mint3 knockdown without affecting the gene expression of HIF1A. There was a trend that HIF-1 target genes VEGFA and HK2 were more suppressed in shMint3#2 compared with shMint3#1, although no statistically significant difference was observed (Figure 2B).

Subsequently, we examined the spheroid formation capacity of these cells and found the reduced spheroid formation capacity by Mint3 knockdown according to the suppression levels, although no statistically significant difference was observed (Figure 2C,D). We further evaluated tumorigenic capacity using a subcutaneous tumor xenotransplantation model in NOD/SCID mice (n = 5). Subcutaneous tumor formation was observed in all mice in the control group, one out of five in the shMint3#1 group, and two out of five in the shMint3#2 group (Figure 2E). The median tumor volume tended to be lower in Mint3 knockdown groups compared with control group (statistical analysis could not be performed due to small group size) (Figure 2F). Therefore, Mint3 knockdown inhibited the tumor-forming capacity of KH cells in vivo. These results suggest that Mint3 plays a fundamental role in the induction of HIF-1 target gene expression during normoxia and tumorigenicity in HCC without affecting the gene expression levels of HIF1A.

### 2.3. Chemical HCC Development Initiated and Promoted by DEN/CCl4 Is Impaired in Mint3 KO Mice

**Figure 3:**
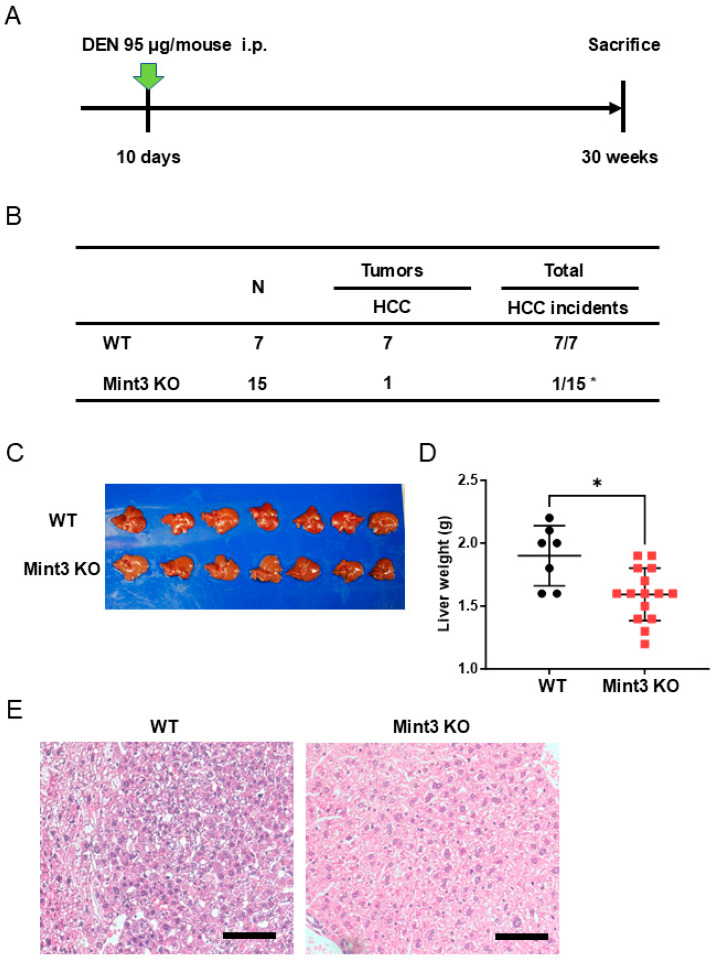
Impairment of DEN-induced hepatocarcinogenesis in Mint3 knockout (KO) mice. (**A**) Administration method and schedule of carcinogen (DEN 95 μg/mouse; intraperitoneal, indicated as green arrow). (**B**) The incidence ratio of HCC in wild-type (WT) and Mint3 KO mice. * *p* < 0.05 by Fisher’s exact test. (**C**) A representative photomicrograph of the liver from WT (n = 7) and Mint3 KO mice (n = 7). (**D**) Median liver weight of WT (n = 7) and Mint3 KO mice (n = 15). * *p* < 0.05 by the Mann–Whitney U tests. (**E**) H&E staining images of the tumor developed in WT (left panel) and Mint3 KO (right panel) mice. Black scale bars in microscopic images indicate 100 μm.

We utilized Mint3 KO and control WT mice to evaluate the role of Mint3 expression on the developmental process of hepatocarcinogenesis. We first intraperitoneally injected DEN, a known chemical carcinogen that induces the initiation of cancer, into mice and analyzed HCC incidence at 30 weeks of age (Figure 3A). The incidence of HCC at 30 weeks was strikingly lower (6.7%) in Mint3 KO mice than in WT mice (100%), without metastasis to other organs in each group (Figure 3B). Consistently, Mint3 KO mice showed decreased liver weight compared with WT mice (Figure 3C,D). Morphologically, tumor cells exhibited high nuclear/cytoplasmic ratios and cell densities in WT mice, whereas such pathological findings were unclear in all tumors that developed in Mint3 KO mice (Figure 3E). These data suggested that Mint3 plays a pivotal role in the development of HCC after DEN initiation.

**Figure 4:**
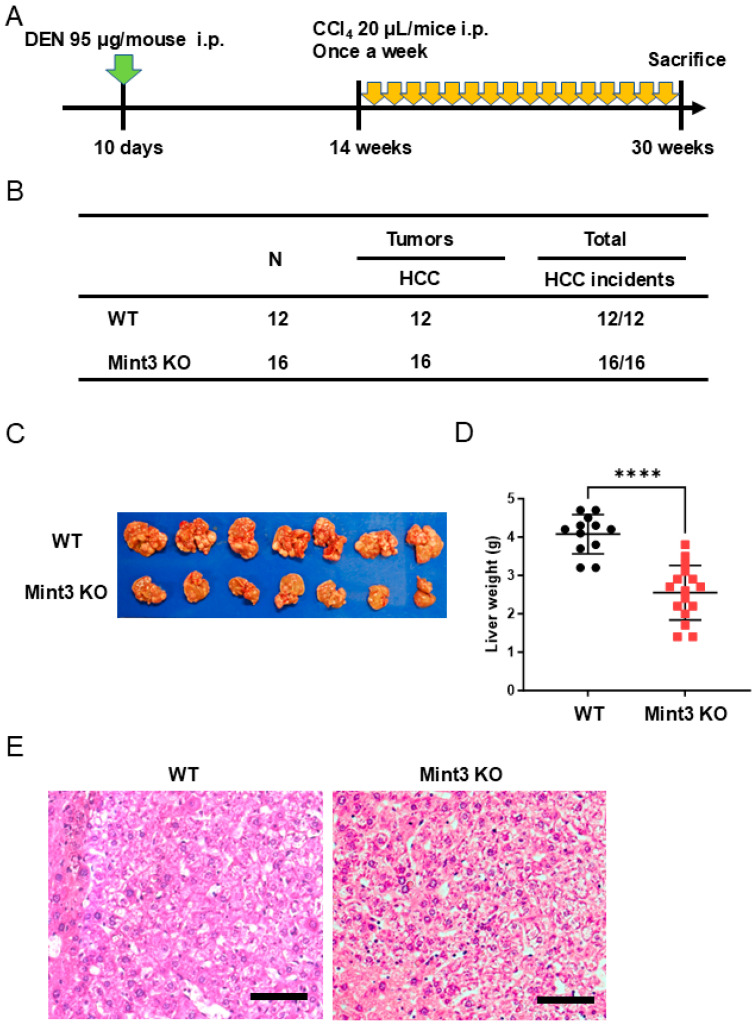
Impairment of DEN/CCl4-induced hepatocarcinogenesis in Mint3 KO mice. (**A**) Administration method and schedule of carcinogen (DEN 95 μg/mouse; intraperitoneal, indicated with green arrow, CCl4 20 μL/mouse; intraperitoneal, indicated with yellow arrows). (**B**) The incidence rate of HCC in WT and Mint3 KO mice. (**C**) A representative photomicrograph of the liver from WT (n = 7) and Mint3 KO mice (n = 7). (**D**) Median liver weight of WT (n = 12) and Mint3 KO mice (n = 16). **** *p* < 0.0001 by the Mann–Whitney U tests. (**E**) H&E staining images of the tumor developed in WT (left panel) and Mint3 KO (right panel) mice. Black scale bars in microscopic images indicate 100 μm.

We further examined the effects of Mint3 expression on DEN-induced HCC development initiated by DEN and promoted/progressed by CCl4 (Figure 4A). In this experimental setting, all WT and Mint3 KO mice showed HCC incidence at 30 weeks of age without distant metastasis (Figure 4B); however, Mint3 KO mice showed decreased liver weight compared to WT mice (Figure 4C,D). Interestingly, all HCCs in WT mice showed moderately differentiated histology with trabecular patterns, whereas well-differentiated cell morphology with fatty changes was observed in all HCCs of Mint3 KO mice (Figure 4E). Since HCC remained well-differentiated in Mint3 KO mice, our data suggest that Mint3 plays a crucial role in the promotion and progression of moderately differentiated HCC.

**Figure 5:**
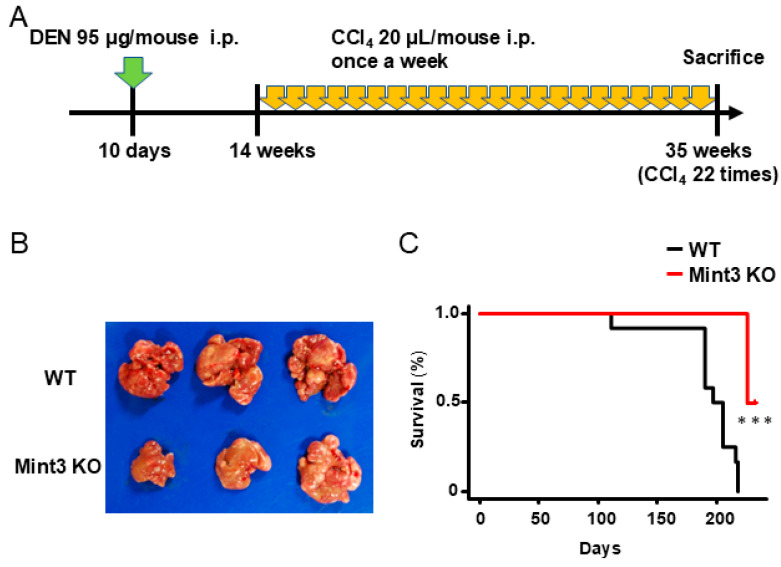
Survival analysis of WT and Mint3 KO mice in DEN/CCl4-induced HCC models. (**A**) Administration method and schedule of carcinogen (DEN 95 μg/mouse; intraperitoneal, indicated with green arrow, CCl4 20 μL/mouse; intraperitoneal, indicated with yellow arrows). (**B**) A representative photomicrograph of the liver from WT (n = 3) and Mint3 KO mice (n = 3) at the endpoint. (**C**) Kaplan–Meier survival curves of WT (black line, n = 12) and Mint3 KO mice (red line, n = 6) in the DEN/CCl4-induced HCC model. *** *p* < 0.001 by the log-rank test.

Finally, we analyzed the effects of Mint3 expression on survival in a DEN/CCl4-induced HCC mouse model (Figure 5A). HCC development at the endpoint was impaired in Mint3 KO mice compared to WT mice (Figure 5B). Consequently, Mint3 KO mice survived significantly longer than WT mice at 35 weeks of age (Figure 5C). Taken together, these data suggest a pivotal role for Mint3 in HCC development at both the promotion and progression stages.

## 3. Discussion

HCC develops in a multistep manner, from dysplastic nodules to well, moderately, or poorly differentiated HCC [7]. Angiogenesis is a hallmark of typical classical moderately differentiated HCC features, radiologically confirmed by the early enhancement of the tumor in the arterial phase in a dynamic CT/MRI study [30]. In contrast, well-differentiated HCCs generally do not display these radiological findings [31]. Nevertheless, tumor cell density is higher in well-differentiated HCC owing to its active cell proliferation compared to dysplastic nodules, and it remains unclear how tumor cells achieve cell growth without angiogenesis, which is generally induced by HIF-1 [32,33]. In this study, we revealed that HIF-1 targets such as VEGFA and HK2 were similarly expressed in hypervascular moderately differentiated and hypovascular well-differentiated regions in a case of nodule-in-nodule HCC. Mint3 is a ubiquitously expressed molecule and has been reported to be expressed in various cancer types, although its expression is reported to be slightly higher in cancer cells than in normal cells [26]. The expression of Mint3 in human HCC tissues at different BCLC stages was investigated using the Chinese Liver Cancer Atlas (CLCA) dataset obtained from cBioPortal (https://www.cbioportal.org (accessed on 3 February 2025); Supplementary Figure S4). Interestingly, Mint3 is frequently overexpressed in the very early stages, typically in well-differentiated HCC. Since Mint3 conveys hypoxia-induced tumor growth signaling without hypoxic accumulation of HIF-1α [22,29], it is plausible that Mint3 activation might be the initial step toward moderately differentiated HCC development from dysplastic nodules or well-differentiated HCC. Indeed, because AFP is typically expressed in moderately to poorly differentiated HCC with a classical hypervascular nature, the inverse correlation between Mint3 expression and serum AFP levels could be attributed to the role of Mint3 in promotion and progression, especially in well-differentiated hypovascular HCC.

The Warburg effect promotes cancer cell proliferation by providing metabolic by-products of glycolysis that are used for cell proliferation [34]. In addition, the Warburg effect has been proposed to reduce excess oxygen consumption via mitochondria, thereby increasing oxygen availability in tumor tissues [35]. Mint3 promotes the Warburg effect, and Mint3 depletion induces severe hypoxia in breast cancer [27,29]. Thus, Mint3 may take advantage of these features of the Warburg effect and contribute to tumor growth even in hypovascular, well-differentiated HCC. In contrast to HCC, Mint3 expression is positively correlated with tumor stage in bladder cancer [24], indicating differential regulation of Mint3 expression depending on cancer origin. The mechanism by which Mint3 expression is regulated in specific types of tumors needs to be addressed using clinical specimens in future studies to clarify the appropriate tumor stage for Mint3 targeting therapy.

Considering the role of Mint3 as an early molecular event in HCC development, Mint3 inhibition may be an ideal molecular strategy for HCC prevention. Indeed, this study demonstrated for the first time the suppressive effect of Mint3 knockdown on spheroid formation and in vivo tumorigenicity in patient-derived well-differentiated HCC cells with preserved hepatocyte function. In the chemical hepatocarcinogenesis model, although the absence of Mint3 seemed tumor-suppressive after both initiation (DEN model) and initiation/promotion (DEN/CCl4 model), its effect was maximized after initiation without promotion treatment (DEN model). Recently, we identified a small molecule, naphthofluorescein, which inhibits Mint3-mediated HIF-1 activation [36]. Administration of naphthofluorescein suppressed tumor growth, chemoresistance, and metastasis in mouse models of several cancer types, similar to the Mint3 inhibition reported in previous studies [25,26,28,34,37]. Mint3 KO mice have been reported to show no obvious developmental abnormalities or survival [38]. Thus, small molecule compounds that inhibit Mint3-mediated HIF-1 activation may be useful for HCC prevention.

There are several limitations in our experiment, and we will discuss our future plans. First, our experiment showed that Mint3 plays a crucial role in early-stage HCC development before hypervascularization by activating HIF-1 target genes before the tumor becomes hypoxic, but these are preliminary data and the detailed mechanism is still under analysis. It is expected that the binding of Mint3 to FIH-1 prevents FIH-1 from suppressing HIF-1, but whether other pathways and signals are involved will be analyzed in the future. Second, our experiment used only Mint3 KO mice, and the overexpression model was not used. Overexpression experiments may allow us to further evaluate the involvement of Mint3 in HCC. However, there are no reports evaluating the effect of Mint3 overexpression in HCC cell lines in vitro or in mouse liver in vivo. There are reports on the expression status of Mint3 in various types of cell lines [27], but Mint3 was basically detected in various cell lines, and different cell lines might have more fundamental genetic differences such as p53/HIF status in addition to the Mint3 status. Clearly, further studies are needed to determine whether these cell lines can be used to create a Mint3 overexpression model. We would like to evaluate the effects of Mint3 overexpression in the tumor as future challenges.

## 4. Materials and Methods

### 4.1. Human HCC Samples

One hundred patients were diagnosed with HCC and underwent surgery at Kanazawa University Hospital. The resected HCC tissues were formalin-fixed, paraffin-embedded, and used for immunohistochemistry as described previously [39]. Clinicopathological information and imaging findings were collected from medical records.

The MRI data of HCC patients treated with 0.025 mmol/kg Gd-EOB-DTPA (Primovist; Bayer Schering Pharma, Berlin, Germany) were obtained at Kanazawa University Hospital. EOB-MRI was performed using the same protocol as described previously [40].

### 4.2. Cell Lines and Reagents

Cells derived from patients with well-differentiated HCC were established as previously described and termed KH cells [40,41]. Briefly, a fresh HCC tissue obtained from a patient with AFP-negative HCC was dissected and digested in 1 mg/mL type 4 collagenase solution (Signa-Aldrich Japan K.K., Tokyo, Japan) at 37 °C for 20 min. Cells were washed twice with ice-cold phosphate-buffered saline and treated with ammonium chloride solution (Stem Cell Technologies Inc., Vancouver, BC, Canada) on ice for 5 min to lyse the red blood cells. The cells were subcutaneously inoculated into nonobese diabetic/severe combined immunodeficiency disease (NOD/SCID) mice. Subcutaneous tumors were dissected, digested, and used for cell culture in vitro.

KH cells with stable Mint3 gene knockdown (shMint3#1 and #2) or control (shLacZ) were established using the pLenti6 BLOCK iT (Thermo Fisher Scientific, Waltham, MA, USA) lentivirus vector as previously described [25]. The sequences of shRNAs were as follows: shLacZ, 5′-GCTACACAAATCAGCGATTTCGAAAAATCGCTGATTTGTGTAG-3′; shMint3#1, 5′-CCGACTGTTGCAGCCCCCTGACGAATCAGGGGGCTGCAACAGTCGG-3′; shMint3#2, 5′-GCGGTTCTTGGTCCTGTATGACGAATCATACAGGACCAAGAACCGC-3′.

### 4.3. Spheroid Formation Assay

A suspension of 2000 KH cells was cultured in a 6-well ultra-low attachment microplate (Corning Inc., Corning, NY, USA). Spheroid formation ability was measured with spheroids greater than 200 µm in diameter 14 days after seeding.

### 4.4. Immunohistochemistry (IHC)

The human and mouse liver tissues were fixed in 10% formalin. Paraffin-embedded tissues were cut at 2 μm thickness and subjected to hematoxylin and eosin and IHC staining. For IHC staining, the paraffin-embedded sections were deparaffinized, rehydrated, antigen retrieval performed by citrate buffer, and blocked by Protein Block Serum-Free (Dako, Carpinteria, CA, USA). The slides were incubated with the following primary antibodies overnight at 4 °C: anti-Mint3 (611380; BD Biosciences, Franklin Lakes, NJ, USA), anti-VEGFA (ab185236; abcam, Cambridge, UK), and anti-HK2 (PA5-29326; protein tech, Rosemont, IL, USA). The slides were processed using an EnVision + Kit (Dako).

Mint3 expression status in Figure 1 was quantified using ImageJ (version 1.54g) [42]. The staining of Mint3, VEGFA, and HK2 in well-differentiated HCC was evaluated and compared to moderately differentiated HCC, indicated as a ratio. Mint3 expression status in surgically resected HCC was also quantified using ImageJ (version 1.54g) [42]. Mint3—positive or negative—was defined as membranous and/or cytoplasmic staining detected in ≦10% of the tumor as score 0, 10%< ~≦50% as score 1, and >50% as score 2, with score 0 being negative and score 1 and 2 being positive (Supplementary Figure S5). In multiple HCC cases, the largest tumor was evaluated as the representative part.

### 4.5. Western Blotting Analysis

Cells were lysed with lysis buffer and centrifuged at 20,000× *g* for 15 min at 4 °C. The supernatants were collected and the total protein content was measured using the Bradford assay (Bio-Rad, Hercules, CA, USA). Lysates were separated by SDS-PAGE, transferred to polyvinylidene fluoride membrane filters, and analyzed by Western blotting using a mouse anti-Mint3 antibody (611380; BD Biosciences, Franklin Lakes, NJ, USA) or a mouse anti-β-actin antibody (MAB1501; Merck Millipore, Burlington, MA, USA). Quantification was performed using ImageJ (version 1.54g) [42] with the following protocol: Kenji Ohgane, Hiromasa Yoshioka 2019. Quantification of Gel Bands by an Image J Macro, Band/Peak Quantification Tool. protocols.io, https://dx.doi.org/10.17504/protocols.io.7vghn3w (accessed on 3 February 2025). We defined the ratio of Mint3 to β-actin in quantified gel band signals as relative expression.

### 4.6. RNA Isolation, Reverse, Transcription (RT), and Quantitative Polymerase Chain Reaction (PCR)

Total RNA was isolated from the cells using an RNeasy Plus Mini Kit (Qiagen, Hilden, Germany) and subjected to RT using ReverTra Ace qPCR RT Master Mix (TOYOBO, Osaka, Japan). The RT products were analyzed by real-time PCR in a 7300HT qPCR system (Applied Biosystems, Foster City, CA, USA) using KOD SYBR qPCR (TOYOBO) and specific primers, as previously described [43]. The expression levels of individual mRNAs were normalized to those of ACTB mRNA.

### 4.7. Animal Models

All animal experiments were approved by the Ethics Committee for the Care and Use of Laboratory Animals, Kanazawa University, Takara-machi Campus, and conducted according to the ARRIVE guidelines 2.0. All experiments were performed in accordance with the relevant guidelines and regulations. For inoculation of patient-derived HCC cells, KH cells were suspended at 1 × 10^6^ cells in 0.2 mL 50% Matrigel (Corning) and subcutaneously transplanted into NOD/SCID mice (Jackson Laboratory Japan, Kanagawa, Japan). Implanted tumors were measured at the endpoint and their volumes were calculated using the formula V = (L × W^2^)/2, where V is the volume (mm^3^), L is the largest tumor diameter (mm), and W is the smallest tumor diameter (mm).

For chemically induced hepatocarcinogenesis models, wild-type (WT) and Mint3 knockout (KO) mice (C57BL/6J background; Riken CDB accession number: CDB0589K) [44] were used to evaluate N-nitrosodiethylamine (DEN)-induced (Sigma–Aldrich, St. Louis, MO, USA) or DEN/CCL4-induced (FUJIFILM Wako, Osaka, Japan) chemical hepatocarcinogenesis. Briefly, mice were intraperitoneally injected with 95 mg DEN at 10 days of age. The mice were then housed under ordinary conditions with a 12-h light/dark cycle. For the DEN/CCl4 model, an additional intraperitoneal injection of 20 mL of CCl4 was initiated 14 weeks after birth and continued every week until sacrifice. The mice were euthanized at 30 weeks of age, and the tumor incidence and morphology were evaluated histologically.

As for the sample size of the DEN model, initially, we started with an equal number of WT and Mint3 KO mice (n = 7). However, the HCC incidence in Mint3 KO mice was significantly lower and no tumor development was detected. To further confirm the experimental accuracy, we doubled the number of Mint3 KO mice to that of the WT mice.

In this experiment, multiple tumors were observed, and as it was difficult to count and calculate the volume of each tumor, liver weight (g) was used for assessment.

### 4.8. Statistical Analysis

Chi-square tests, *t*-tests, Mann–Whitney U tests, and Kaplan–Meier survival analyses with log-rank tests were performed using Prism 7 (GraphPad Software, San Diego, CA, USA). Statistical significance was set at *p* < 0.05.

## 5. Conclusions

In conclusion, Mint3 was overexpressed in well-differentiated HCC, and Mint3 suppression attenuated the in vitro spheroid formation and tumorigenicity of patient-derived well-differentiated HCC cells. In addition, Mint3 depletion prevented the incidence of HCC in chemically induced mouse hepatocarcinogenesis models. Thus, molecular targeting of Mint3 function using small molecules may pave the way for the development of novel HCC preventive medicines in the future.

## Figures and Tables

**Table 1:** Clinicopathological characteristics of 100 consecutive surgical cases by Mint3 expression status.

Parameters	Mint3 Negative	Mint3 Positive	*p* Value
	(n = 56)	(n = 44)	
Age (year, mean ± SE)	64.7 ± 1.3	65.3 ± 1.6	0.775
Sex (male/female)	40/16	32/12	1
Etiology (HBV/HCV/B + C/other)	15/19/20/2	8/18/18/0	0.519
AFP (ng/mL, mean ± SE)	3830 ± 1366	364.6 ± 217.5	0.0285
PIVKAII (mAU/mL, mean ± SE)	4201 ± 1530	2751 ± 1633	0.521
AST (IU/L, mean ± SE)	42.9 ± 4.09	41.3 ± 2.87	0.761
ALT (IU/L, mean ± SE)	37.1 ± 3.54	38.9 ± 2.60	0.691
Plt (×10^3^, mean ± SE)	139 ± 7.26	152 ± 12.1	0.316
LC/non-LC	27/29	25/19	0.426
Histological grade of HCC			
Well-differentiated	7	14	
Moderately differentiated	38	28	
Poorly differentiated	11	2	0.0128
Tumor size (<5 cm/>5 cm)	36/20	33/11	0.282

## Data Availability

The original contributions presented in this study are included in the article/Supplementary Materials. Further inquiries can be directed to the corresponding authors.

## Supplementary Materials

The following supporting information can be downloaded at: https://www.mdpi.com/article/10.3390/ijms26041430/s1.
