# Supplementary material for: Mint3 as a Molecular Target Activated in the Early Stage of Hepatocarcinogenesis
Source: Int J Mol Sci. 2025 Feb 8;26(4):1430. doi: 10.3390/ijms26041430 (PMC11855386; doi:10.3390/ijms26041430)
Supplement: Supplementary file 1 [file ijms-26-01430-s001.zip › ijms-3444112-supplementary.pdf]

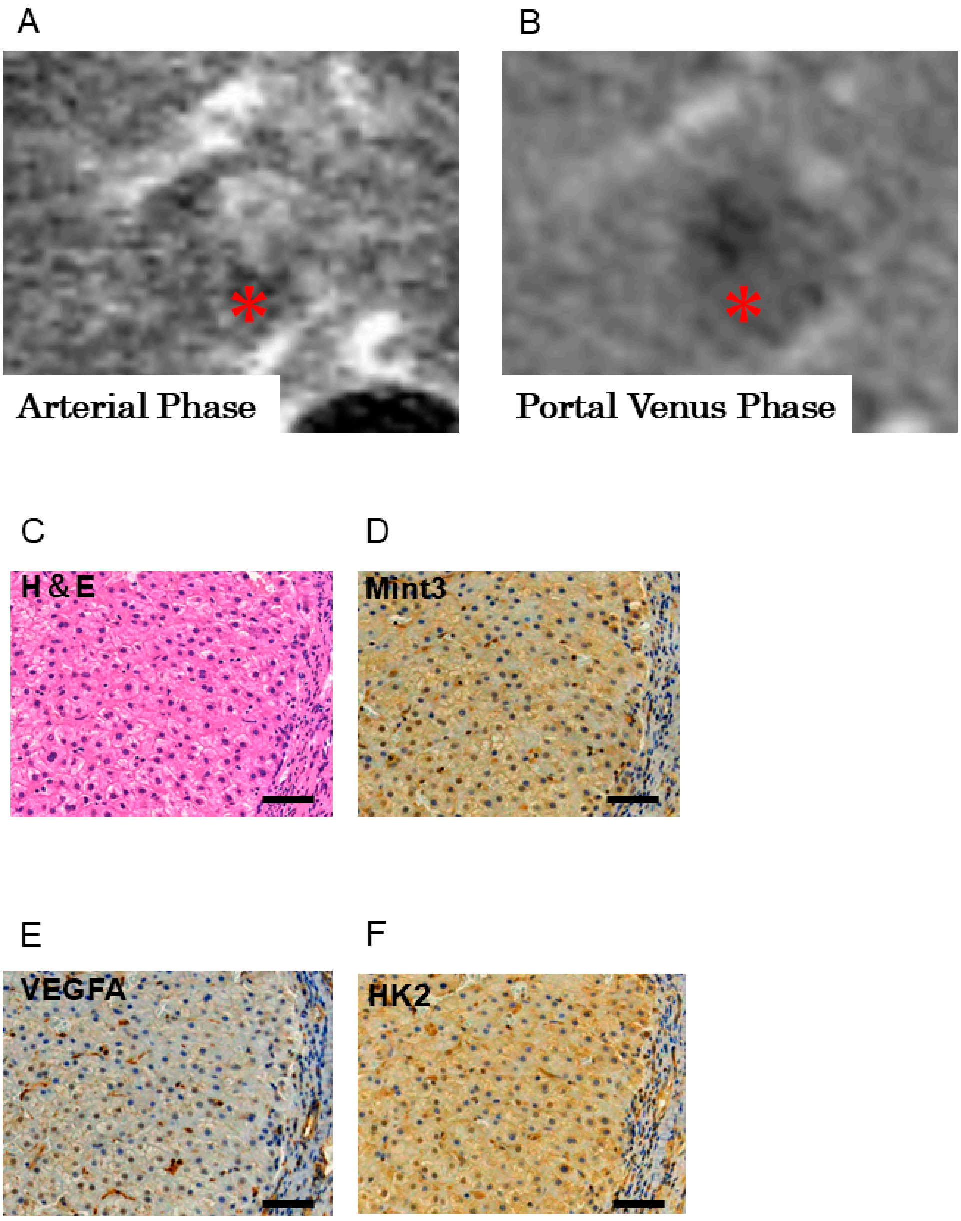


**Supplementary Figure S1.** Expression of Mint3 and HIF-1 target proteins in nodule in nodule HCC (case 2). (A, B) Multiphasic multidetector-row CT images: (A) arterial phase; (B) portal venous phase. Red asterisk (A,B) indicates well-differentiated HCC region with isoattenuation during the arterial and portal venous phases. (C) Microscopic image of red asterisk stained with hematoxylin and eosin. (D-F) Immunohistochemistry analysis of Mint3 (D), VEGFA (E), and HK2 (F) expression in HCC. Black scale bars in microscopic images indicate 100μm.

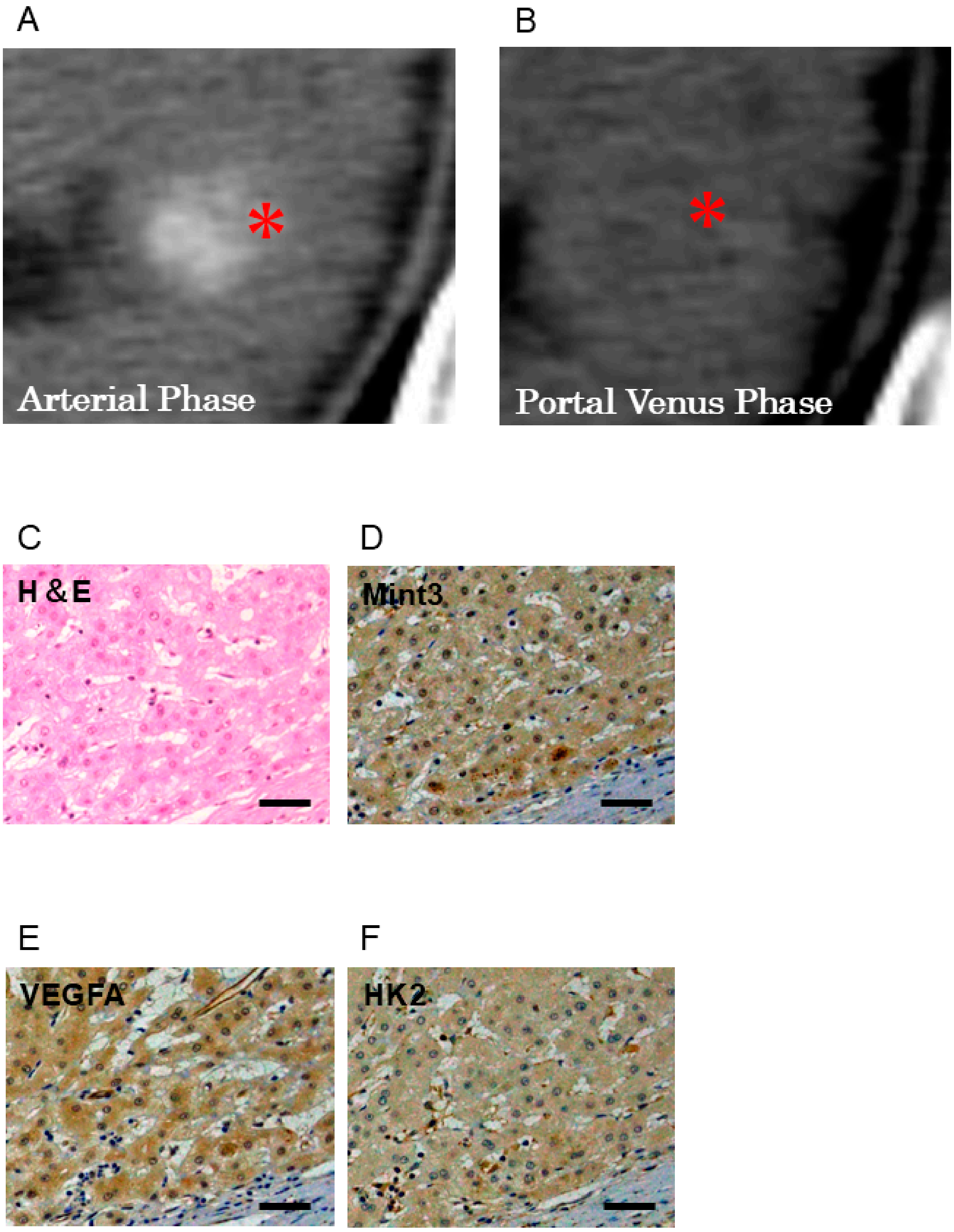


**Supplementary Figure S2.** Expression of Mint3 and HIF-1 target proteins in nodule in nodule HCC (case 3). (A, B) Multiphasic multidetector-row CT images: (A) arterial phase; (B) portal venous phase. Red asterisk (A,B) indicates well-differentiated HCC region with hyper-attenuation and isoattenuation during the arterial and portal venous phases. (C) Microscopic image of red asterisk stained with hematoxylin and eosin. (D-F) Immunohistochemistry analysis of Mint3 (D), VEGFA (E), and HK2 (F) expression in HCC. Black scale bars in microscopic images indicate 100μm.

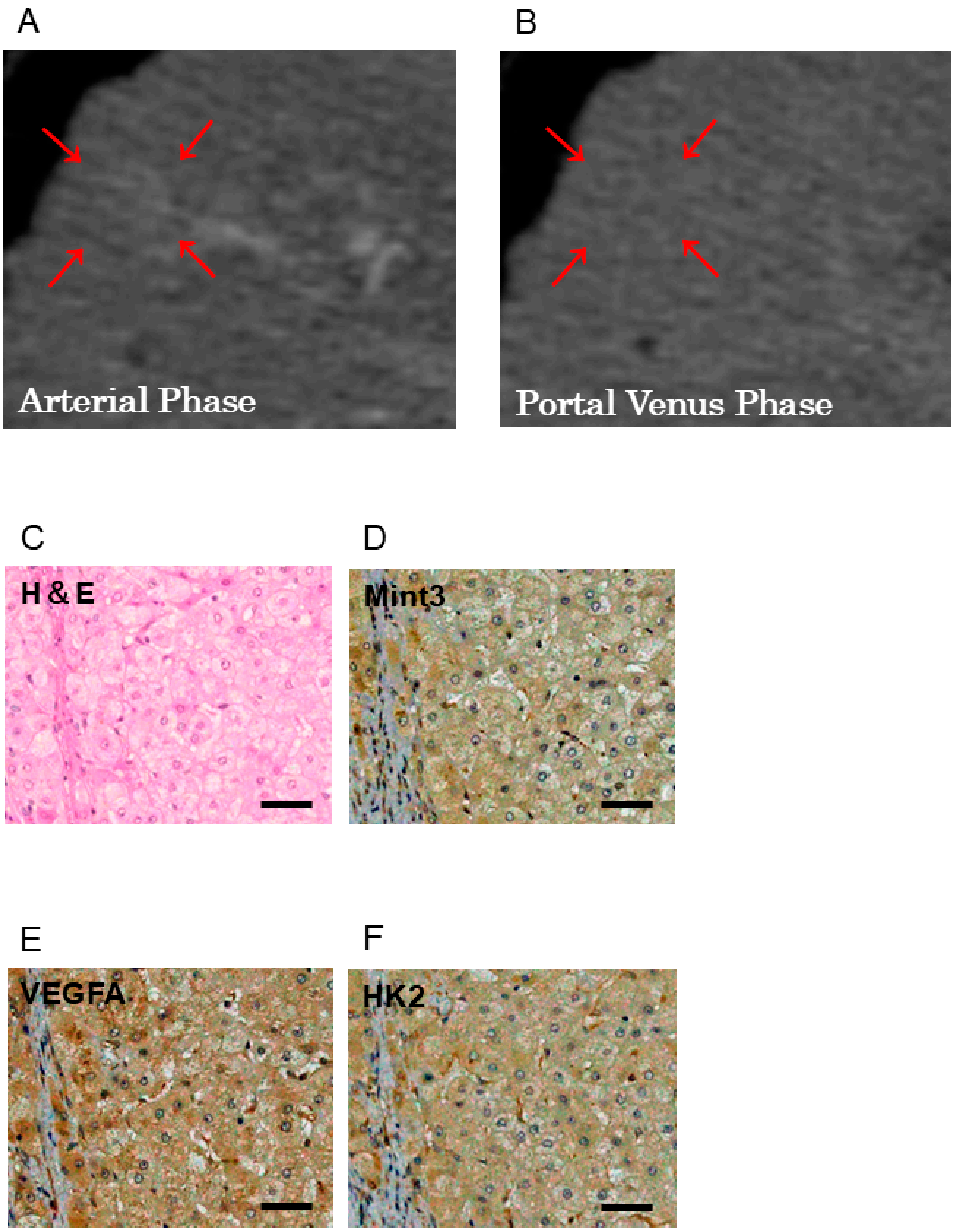


**Supplementary Figure S3.** Expression of Mint3 and HIF-1 target proteins in early well-differentiated hepatocellular carcinoma.(A, B) Multiphasic multidetector-row CT images: (A) arterial phase; (B) portal venous phase. Red arrow (A,B) indicates early HCC region with hypo-vascularity. (C) Microscopic image stained with hematoxylin and eosin. (D-F) Immunohistochemistry analysis of Mint3 (D), VEGFA (E), and HK2 (F) expression in HCC. Black scale bars in microscopic images indicate 100μm.References.

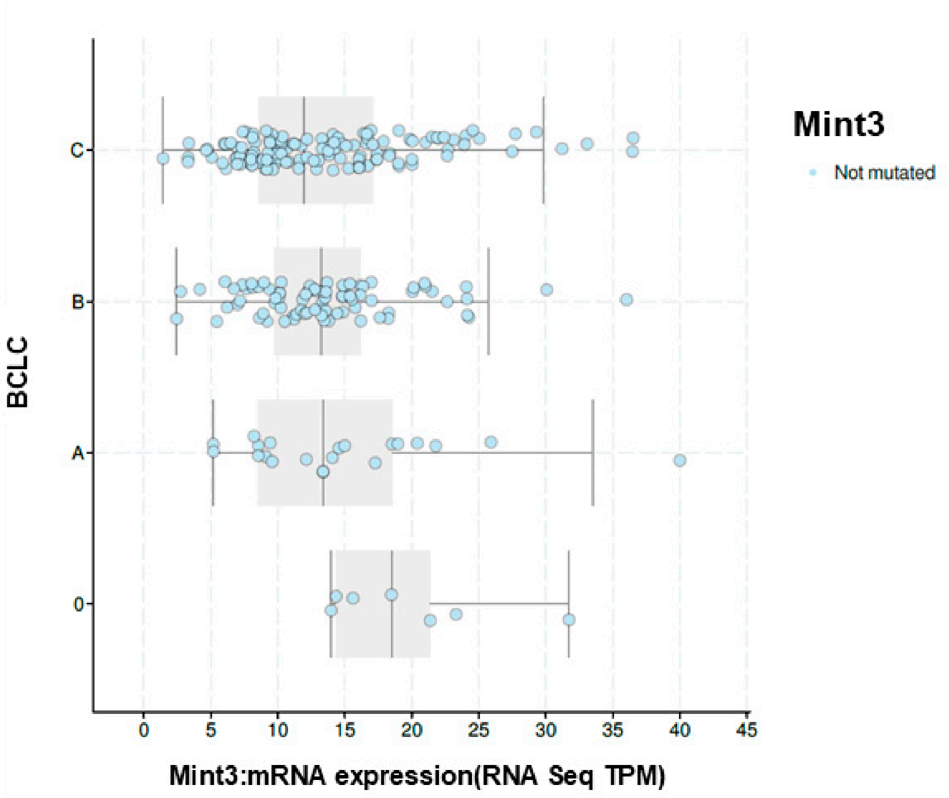


**Supplementary Figure S4. The expression of Mint3 in human HCC tissues at different BCLC stages. Mint3 expression tends to be higher in those with lower BCLC stage. We were investigated using the Chinese Liver Cancer Atlas (CLCA) dataset obtained from cBioPortal (https://www.cbioportal.org ; accessed on 3rd February, 2025).**

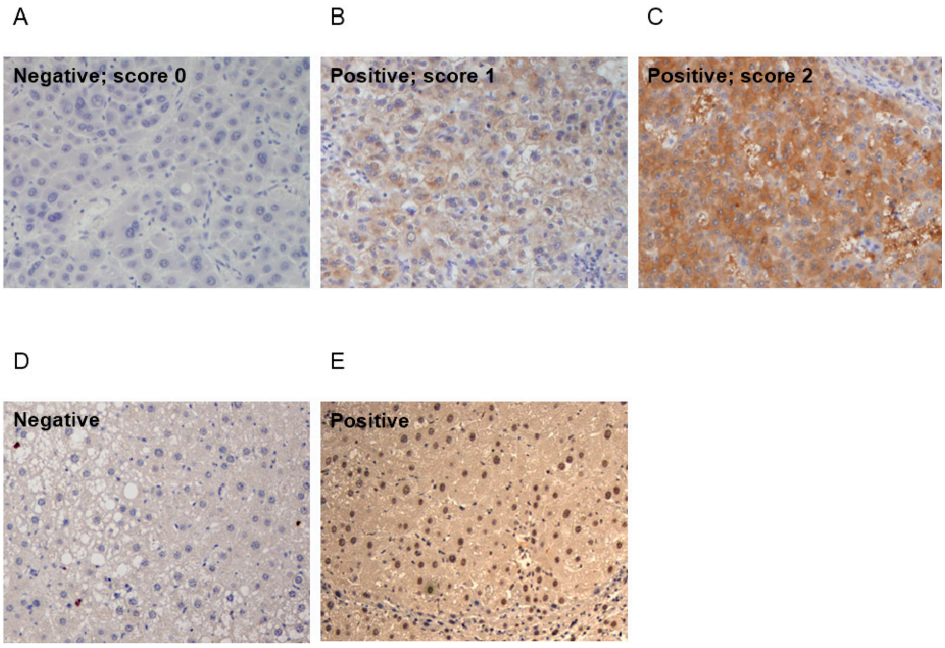


**Supplementary Figure S5. Representative staining of Mint3 in both human-derived tumors (A, B, C) and mouse-derived tumors (D, E). The Staining status of Mint3 was defined as follows: (A) negative with score 0, (B) positive with score 1, (C) positive with score 2 (analyzed using ImageJ). Mouse-derived tumors were determined only negative (D) and positive(E).**
